# Surveillance Colonoscopies of Synchronous Colorectal Cancer: What Should We Do?

**DOI:** 10.5152/tjg.2022.22056

**Published:** 2023-03-01

**Authors:** Chunmei Guo, Jing Wu, Yadan Wang, Hui Su, Canghai Wang, Hong Liu

**Affiliations:** 1 Gastroenterology Department, Beijing Shijitan Hospital, Capital Medical University, Beijing, China; 2 Gastroenterology Department, Beijing Friendship Hospital, Capital Medical University, Beijing, China

**Keywords:** Clinicopathological characteristics, metachronous adenoma, metachronous advanced adenoma, surveillance colonoscopies, synchronous colorectal cancer

## Abstract

**Background:**

The aim of this study is to investigate the occurrence of metachronous neoplasms at 2-year surveillance colonoscopy for synchronous colorectal cancer patients and the relative risk factors.

**Methods:**

Synchronous colorectal cancer patients who underwent surgery or endoscopic resection for colorectal cancer between January 2008 and December 2019 were enrolled. All patients underwent surveillance colonoscopies at least twice within 2 years after operation. Univariate and multivariate analyses were conducted to assess the risk factors for the metachronous neoplasms.

**Results:**

Totally 38 patients (male/female: 26/12) were included, with an average age of 64.6 years (±11.5 years) and a mean surveillance interval of 23.47 ± 4.39 months. In 21 of 38 patients (55.3%), metachronous adenoma was detected, including 6 metachronous advanced adenomas. Two patients were detected with metachronous carcinomas. In univariate analysis, male sex, elderly age at diagnosis, and the presence of synchronous adenomas/synchronous advanced adenoma at baseline colonoscopy were associated with the development of metachronous adenoma (*P* = .037, .047, .013, .039), but not associated with metachronous advanced adenoma (*P* = 0.455, .746, .503, .269). Patients tends to occur less metachronous advanced adenoma if index colorectal tumors were treated by endoscopic resection (*P* = .010), but the tendency was not discovered in metachronous adenoma (*P* = .289). Tumor location (with/without rectum cancer) was not associated with the development of metachronous lesions (*P* = .526, .382). On multivariate analysis, the presence of synchronous adenomas at baseline colonoscopy was an independent risk factor for MA during follow-up (odds ratio = 15.0; 95% CI: 1.55-145.22).

**Conclusion:**

For postoperative synchronous colorectal cancer patients, doctors should design individual surveillance strategies according to sex, baseline colonoscopy, and operative (or endoscopic) approach of resection.

**Main Points**

- Combining endoscopic resection and surgery could improve the discovery rate of synchronous colorectal cancer patients (SCRC) and offers significant surveillance colonoscopy benefit.
- Patients with SCRC in the presence of synchronous neoplasms should undergo follow-up colonoscopy more closely for the early detection of metachronous neoplasms.
- Individual surveillance strategies should be designed for postoperative SCRC patients according to sex, baseline colonoscopy, and approach of resection.

## Introduction

Colorectal cancer (CRC) is the third most common cancer worldwide.^1^ Synchronous colorectal carcinoma (SCRC) refers to more than 1 primary colorectal carcinoma detected in a single patient at initial presentation. The prevalence of SCRC is approximately 3.5% of all colorectal carcinomas.^2^ Over recent years, with the improvement of colonoscopy technology, many early-stage colorectal cancers were diagnosed and treated by endoscopy (endoscopic mucosal resection [EMR] or endoscopic submucosal dissection [ESD]). Studies showed that, different from solitary colorectal carcinoma, SCRC was significantly associated with poor prognosis.^2^ Though postoperative endoscopic surveillance has become a routine part of clinical practice, studies are lacking for those SCRC patients. Considering that most epidemiological studies indicate a higher incidence of metachronous adenoma (MA) in the initial 2-3 years following surgery, and significantly decreased thereafter,^3,4^ we aimed to assess the occurrence of metachronous neoplasms at 2-year surveillance colonoscopy for SCRC patients and the relative risk factors.

## MATERIALS AND METHODS

### Study Population

In this retrospective and prospective cohort study, SCRC patients who underwent surgery or endoscopic treatment (EMR or ESD) for colorectal cancer (CRC) between January 2008 and December 2019 were enrolled. The study was approved by the ethical committee of Beijing Shijitan Hospital, Capital Medical University (No: sjtkyll-lx-2021(49) and informed consent was collected from the patients. All patients had undergone (1) a complete preoperative colonoscopy with cecum intubation for non-obstructing tumors or index colonoscopy within 6 months after curative resection for obstructing tumors; (2) clearance of all synchronous lesions; (3) twice follow-up surveillance colonoscopies (an interval of ≥6 months) in 24 months after surgery.

Patients were excluded for these reasons: (1) patients diagnosed with inflammatory bowel disease, familial adenomatous polyposis, or Lynch syndrome; (2) patients who underwent colectomy for other diseases; (3) patients with poor bowel preparation at the baseline or surveillance colonoscopy; (4) patients with incomplete medical records. Poor bowel preparation was defined as “poor” or “inadequate” according to the Aronchick Bowel Preparation Scale or if the Boston score was <2 for each colonic segment.^5,6^

Synchronous colorectal carcinoma was defined as 2 or more histologically distinct CRCs that are diagnosed simultaneously or in a period less than 6 months after the first diagnosis.^7^ When multiple tumors are located in the same colon segment, lesions should be distinctly separated by at least a 4-cm distance between each other and should not consist of submucosal spread. In synchronous cases, index lesions were defined as the most pathologically advanced tumors or as the largest lesion when 2 or more lesions were in an identical pathological stage.^8^

### Colonoscopic and Histological Examinations

All colonoscopic examinations were performed by professional endoscopists (accomplished more than 1000 colonoscopies individually) using the EVIS LUCERA CV-260 or CV-290 colonoscope (Olympus Medical Systems, Tokyo, Japan). All detected polyps were removed via biopsy, EMR, or ESD, and the specimens were histologically assessed by experienced pathologists. The size of each polyp was estimated using open biopsy forceps or measured directly after polypectomy.

Synchronous adenomas (SA) and synchronous advanced adenomas (SAA) were defined as adenomas or advanced adenomas that are discovered in baseline colonoscopy. A baseline colonoscopy was defined as a preoperative colonoscopy for non-obstructing tumors and as colonoscopy at 6 months after curative resection for obstructing tumors,^5^ while advanced adenomas were defined as high-grade dysplasia of tubular and serrated adenomas, adenomas with at least 25% villous component, and adenomas larger than 10 mm according to the United States Multi-Society Task Force guidelines.^9^

### Data Collection

The following clinical traits, cancer-related variables, and baseline colonoscopy data were collected: gender, age, preoperative carcinoembryonic antigen (CEA) level, comorbidities, tumor location (tumor located from cecum to splenic flexure were defined as right-sided colon cancer, while tumor located from splenic flexure to sigmoid was defined as left-sided colon cancer), tumor stage, tumor pathology (differentiation rate, mucinous component, lymphatic invasion, venous invasion, nervous invasion), and baseline colonoscopy data (presence or not of SA/SAA). Tumor staging was based on the eighth edition of the American Joint Committee on Cancer Board staging criteria.^10^

### Outcome Measures

The primary outcome of this study was the occurrence of MA at the 2-year surveillance colonoscopy after operation, while the secondary outcome was the occurrence of metachronous advanced adenomas (MAA).

### Statistical Analyses

All the statistical analyses were performed by using Statistical Package for the Social Sciences (SPSS) 20.0 (IBM Corp.; Armonk, NY, USA). Continuous variables were expressed as mean ± standard (mean ± SD) and categorical variables as frequencies or percentages. Based on clinical knowledge and the literature, potential baseline risk factors associated with the detection of metachronous neoplasia at follow-up evaluation were selected. Univariate analysis was conducted with the independent-sample *t*-test and the χ^2^-test, as appropriate. Multivariate analysis was performed with logistic regression to assess the independent risk factors for MA (MAA), and the adjusted odds ratio (OR) along with 95% CIs were calculated.

## Results

### Patient Characteristics

**Figure 1.**
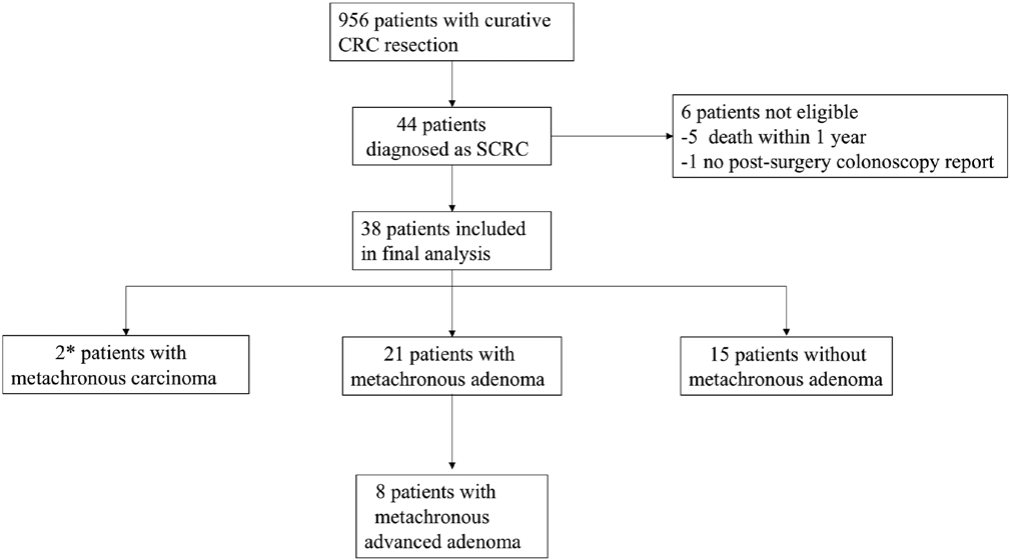
Flowchart of included patients. *1 patient detected MC at 9 months after surgery; 1 patient detected MC at 17 months after surgery. CRC, colorectal cancer; SCRC, synchronous colorectal cancer; MC, metachronous carcinoma.

Of the 956 patients who underwent curative CRC resection, 44 patients were diagnosed with SCRC (4.6%, 44/956). During surveillance, 6 patients were excluded because of death or dropping out. In the final cohort, 38 patients (male/female: 26/12) were included, with an average age of 64.6 years (±11.5 years) and a mean surveillance interval of 23.47 ± 4.39 months (interquartile range: 9-30 months) (Figure 1). Comorbidities were as follows: hypertension (21 cases), diabetes mellitus (7 cases), hyperlipidemia (9 cases), and coronary heart disease (5 cases).

Of the SCRC patients, the majority (n = 33, 86.8%) had 2 tumors (a total of 66 tumors), while 5 patients had 3 tumors (a total of 15 tumors). Table 1 shows the anatomical distribution and stage of the index and concurrent lesions of patients with SCRCs.

In 23 patients, all tumors (2 tumors or 3 tumors) were localized in the colon (48/81 tumors, 59.3%), of which 10 patients were in 1 colon segment (18/81 tumors, 22.2%). In 15 patients, tumors were situated in the rectum (16/81 tumors, 19.8%), of which 1 patient was diagnosed with 2 rectal cancers.

### Comparison Between Index Lesions and Concurrent Lesions

Most patients (n = 32, 84.2%) were diagnosed with III/IV stages in the index tumor, while 5 patients were diagnosed with I/II stages. Index lesions were more frequently moderately differentiated, more deeply penetrated, and more often ulcerated in morphology than concurrent lesions (Table 2). In total, 19 tumors were diagnosed as stage T1 or Tis, of which 11 tumors (57.9%) were resected under colonoscopy, with a curative resection rate of 100%.

### Type of Surgery for Synchronous Colorectal Carcinoma

**Figure 2.**
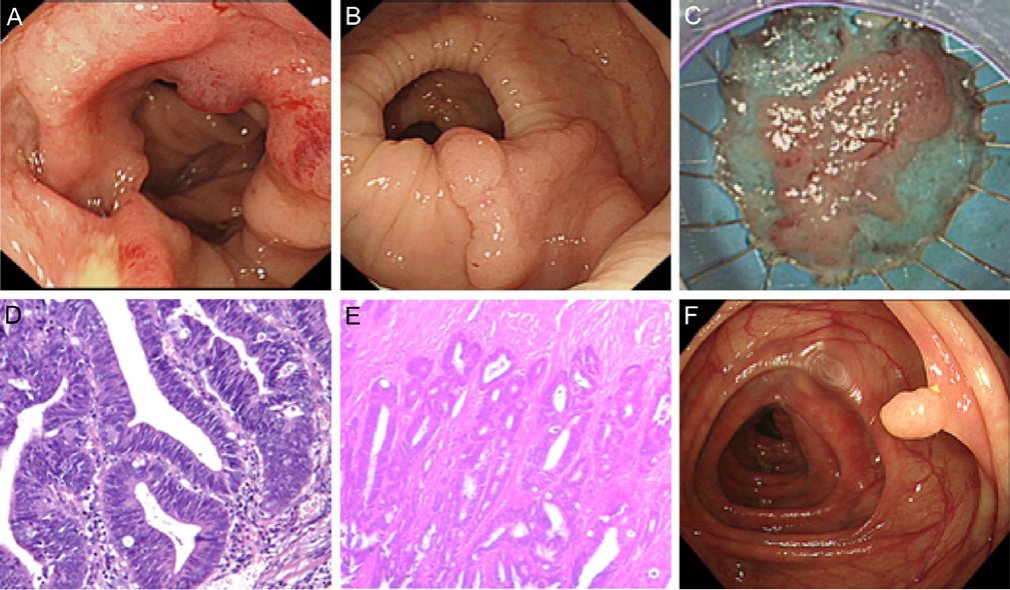
Surgical resection for colon tumor and ESD for LST in 1 SCRC patient. A male patient, 70 years old. (A) 4 × 5 cm mass was observed in hepatic flexure. (B) A 3.0 cm × 2.0 cm LST was observed in splenic flexure under the endoscope; (C) ESD of the LST with Dualknife before surgery, and the specimen was paved; (D) The LST’s pathology shows high-grade intraepithelial neoplasia and focal canceration with negative horizontal resection margins. (E) Ten days after ESD, the hepatic flexure mass was resected under surgery, and its postoperative pathologyshows moderately differentiated adenocarcinoma. (F) A 0.6 × 0.6 cm polyp were observed in survelliance colonoscopy, pathology diagnosed as tubular adenoma. ESD, endoscopic submucosal dissection; LST, laterally spreading tumor; SCRC, synchronous colorectal cancer.

**Figure 3.**
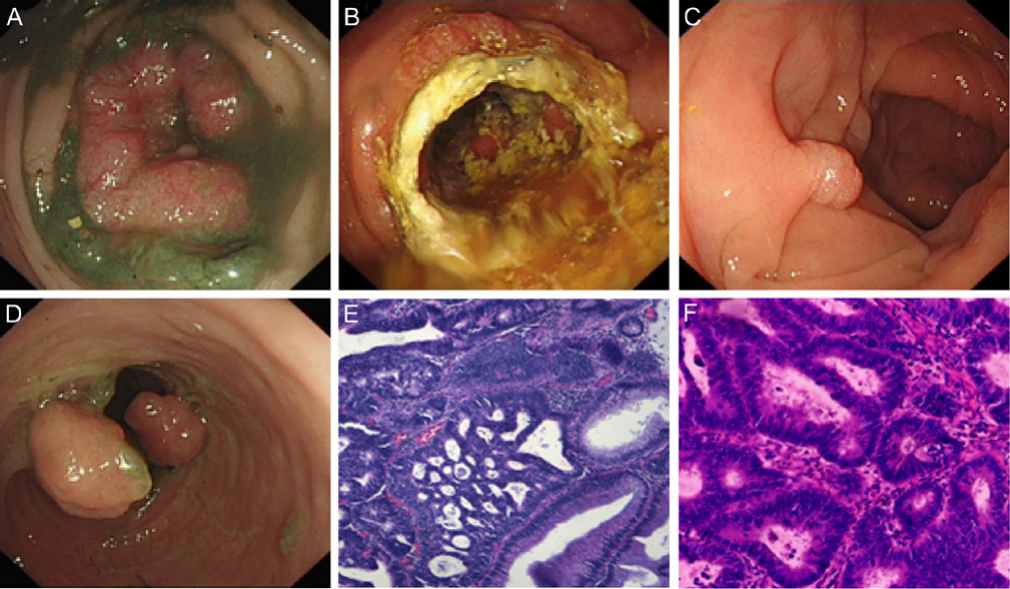
Self-expanding metallic stent as a “bridge to surgery” for obstructive colon cancer and EMR for earlier-stage cancer in one SCRC patient. A male patient, 72 years old. (A) Colonoscopy reveals a bulky tumor at the splenic flexure, a standard colonoscope or gastroscope cannot through. (B) A SEMS was placed through the stenosis. (C) One week later, colonoscope pass through the tunnel without resistance and reach to the cecum. A 0.8 × 0.8 cm polyp was observed in ascending colon and resected by EMR. (D) Another two 1.5 × 1.5 cm polyp were observed in sigmoid colon (at 15 cm above the anus) and resected by EMR too. (E) The sigmoid polyp’ pathology shows high-grade intraepithelial neoplasia and focal canceration with negative horizontal resection margins. (F) The bulky tumor was operated removed 1 week after EMR. Its postoperative pathology shows highly differentiated adenocarcinoma. SEMS, self-expanding metallic stent; EMR, endoscopic mucosal resection; SCRC, synchronous colorectal cancer.

Synchronous colorectal carcinoma patients often required resection of multiple segments (right-left colon 50.0%; right colon-rectum 20.0%; left colon-rectum 30.0%) or extended resection (subtotal colectomy 7.7%; right extended hemicolectomy 26.9%; left extended hemicolectomy 15.4%; extended transverse colectomy 7.7%; sigmoid resection 7.7%; low anterior resection 34.6%). Nine patients (11 tumors) were curatively resected by colonoscopy. Endoscopic resection (ER) was more often performed for bilateral colon tumors (77.8%), most commonly en-bloc resection by endoscopic detection. Figures 2 and 3 show surgical resection and endoscopic resection in SCRC patients.

### Baseline and Surveillance Colonoscopy

In 28 of 38 patients (73.9%), preoperative baseline colonoscopy was performed for non-obstructing tumors. In contrast, approximately a quarter of the patients (10/38, 26.3%) were referred for baseline colonoscopy at 6 months after curative resection of obstructing tumors. Among all cases, 30 SCRCs were diagnosed with SA and 24 were diagnosed with SAA. The median number of SAs was 5 (range 1-20). Preoperative CEA levels were associated with the detection of SA (*P* = .047). However, this correlation was not detected in SAA (*P* = .108). We did not find an association between medical illness (hypertension, diabetes mellitus, hyperlipidemia, and coronary heart disease) and SA.

In 21 of 38 patients (55.3%), MA was detected, including 6 MAA. Two patients had MC (1 patient had MC detected at 9 months after surgery and 1 patient had MC detected at 17 months after surgery). The mean number of MA and MAA per patient followed was 2.69 and 0.31, respectively, and men’s MA detection rate was significantly higher (*P* = .034).

### Risk Factors for Metachronous Neoplasms

Table 3 shows the predictors of MA and MAA during the follow-up. In univariate analysis, male sex, elderly age at diagnosis, and the presence of SA/SAA at baseline colonoscopy were associated with the development of MA (*P* = .037, .047, .013, .039). However, they were not associated with MAA (*P* = .455, .746, .503, .269). Male sex was associated with a higher incidence of MA (70.8% in men vs. 33.3% in women, *P* < .05). On the other hand, CEA level, tumor location (with/without rectal cancer), and stage of index tumor (Tis/I/II vs. III/IV) were not associated with the development of metachronous lesions (MA or MAA) (*P* = .187, .526, .185; *P* = .302, .382, .382, respectively).

Eleven tumors (9 patients) were curatively resected by colonoscopy. The relationship between ER (which means EMR or ESD) and metachronous lesions was inconsistent. Patients tended to have less MAA if index CRCs were treated by ER (*P* = .010), but the tendency was not discovered in MA (*P* = .289).

On multivariate analysis, the presence of SA at baseline colonoscopy was an independent risk factor for MA during the follow-up (OR = 15.0; 95% CI: 1.55-145.22), which means that there was a trend for a higher rate of development of MA for those patients.

### Radiological Examination

All patients underwent enhanced computed tomography (CT) in addition to colonoscopy in the postoperative period. Distant metastasis was found in 9 (23.7%) patients during the follow-up, including 2 cases of hepatic metastasis, 3 cases of pulmonary metastasis, and 4 cases of lymph node metastasis. However, we did not find a correlation between radiological findings and the follow-up surveillance colonoscopy.

## Discussion

The present study collected the clinicopathological data and 2-year postoperative endoscopic surveillance, investigating for the relative risk factors of metachronous neoplasms in SCRC patients.

Lam et al^2^ reported an overall SCRC incidence of 3.5% (3667/105686) based on the data from 39 studies (ranged from 1.1% to 8.1%, neither in Asia nor in Europe). The prevalence of SCRC was 4.6% in the current study, consistent with previous studies. Synchronous colorectal carcinoma was more often seen in men,^11^ the male to female ratio was 1.8 : 1.0 (2260 men vs. 1241 women) based on 38 studies.^2^ In this study, the sex ratio was 2.17 : 1 (male/female: 26/12), similar to the literature. In univariate analysis, the male sex was associated with the development of MA (*P* = .037), but not associated with MAA (*P* = .455). Male sex tends to have a higher incidence of MA (70.8% in men vs. 33.3% in women, *P* < .05). However, male sex was not an independent risk factor for MA on multivariate analysis. Large-scale studies will be required to verify the relationship between male sex and metachronous lesions.

By pooling the data from 32 series in analysis, the mean age at SCRC diagnosis was 63 years (ranged from 47 to 79 years) in Lam’s study.^2,12^ In the current study, the age at diagnosis for SCRC was 64.6 years (±11.5 years), similar to the present research. Elderly age at diagnosis was associated with the development of MA (*P* = .047) but was not an independent risk factor. This demonstrated that surveillance colonoscopy should be carried out regardless of age.

Carcinoembryonic antigens were useful prognostic factors in CRC.^13^ Compared to solitary CRC, abnormal CEA was more commonly seen in patients with SCRC.^14^ Preoperative CEA positivity was a significant predictor of SA for SCRC patients (*P* = .047) but was not associated with metachronous lesions (*P* = .187, .302, respectively). Endoscopists should pay more attention to preoperative colonoscopy for those SCRC patients with abnormal CEA.

The anatomical distribution of SCRC is inconsistent in different reports.^2,15,16^ In some studies, SCRC occurred in the same segment or close to each other; nevertheless, some SCRCs are noted in different segments of the large intestine in the other studies.^11^ Lam et al^2^ considered the common sites of SCRC as the sigmoid colon or rectum. In this study, 22.2% (18/81) tumors occurred in the same segment of the colon, while 19.8% (16/81) tumors were situated in the rectum. However, tumor location (with/without rectum cancer) was not significantly associated with the development of metachronous lesions (MA or MAA) (*P* = .526, .382). Considering the wide distribution of SCRC, thorough preoperative colonoscopy was very important, without regard for the location of SCRC.

Studies have shown that some tumor morphology is more common in patients with synchronous tumors.^2,16,17^ Index tumors of synchronous cases were more frequently moderately differentiated, mucinous adenocarcinoma, more deeply penetrated, and more often ulcerated, while concurrent lesions were less advanced.^18^ Our results reproduced Oya et al’s^18^ report. In this study, 32 out of 38 index lesions were III/IV stage, but the stage of index tumor (Tis/I/II vs III/IV) was not associated with the development of metachronous lesions (MA or MAA) (*P* = .185, .382). Therefore, we suggested a close colonoscopy follow-up, no matter what stage of index lesions.

**Figure 4.**
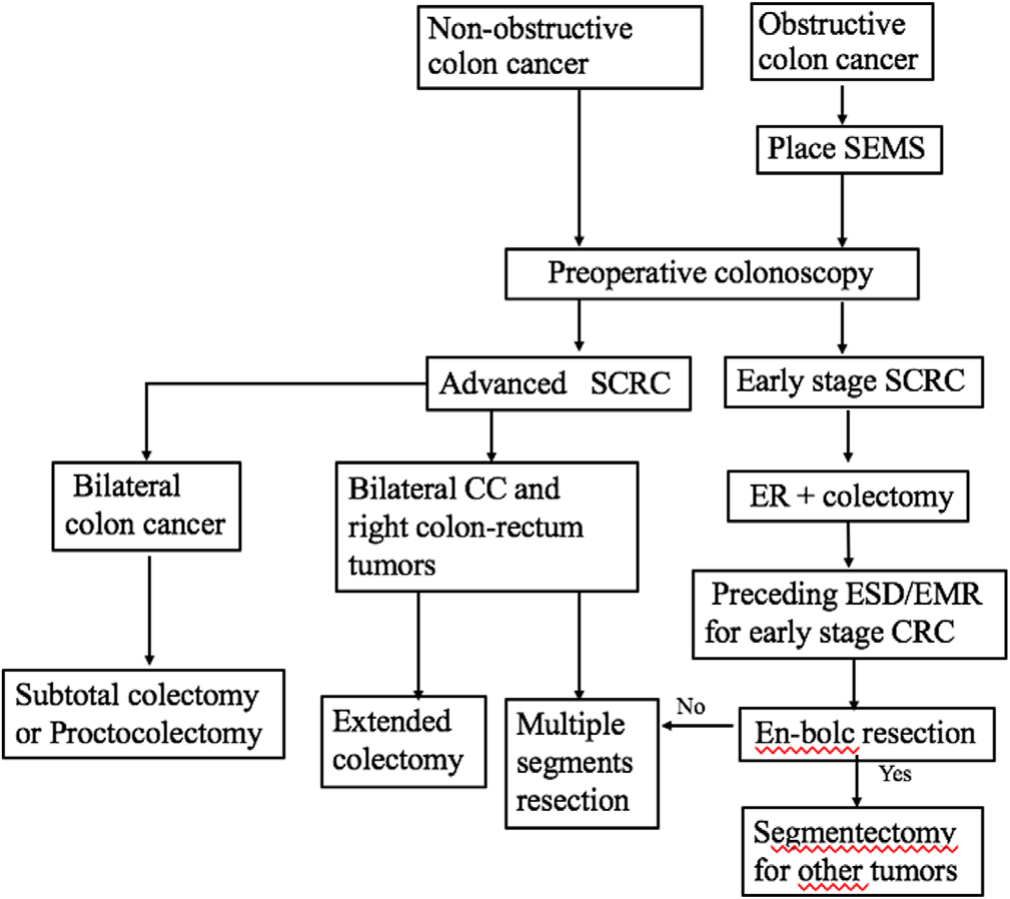
The treatment strategy in patients with SCRC. SCRC, synchronous colorectal cancer; SEMS, self-expanding metallic stent; ER, endoscopic resection; ESD, endoscopic submucosal dissection; EMR, endoscopic mucosal resection; CC, colon cancer; advanced SCRC, all colorectal tumors were diagnosed as advanced stage; early stage SCRC, at least one tumor was diagnosed as early stage.

For SCRC that is distributed in multiple segments, the required extent of the procedure for tumors has remained controversial in the past decades.^17,19^ Existing research demonstrated that there is no benefit in the survival of subtotal colectomy; therefore, segmental resection should be performed whenever possible.^20^ In our study, most patients underwent multiple segment resection or extended resection, consistent with the previous studies.^17^ Population-based studies have shown that colorectal neoplasms without signs of deep submucosal invasion can be treated by ER (ESD or EMR), which could reduce the incidence and mortality of CRC. In a multicenter study conducted in Japan, colorectal polyps (2 cm and larger) were removed by ER, 9.9% of which were staged as T1. In our study, 11 (16.2%) T1/Tis tumors were treated by ER with an en-bloc resection rate of 100%. Interestingly, although ER was not associated with MA, more ER indicated less MAA (*P *< .05). In a propensity-score-matched study, Yamashita et al analyzed the influence of preceding ESD combined with surgical operation on the prognosis of T1 stage colorectal cancer. In these patients, preceding ESD with histological en-bloc resection did not affect their post-operational oncologic behavior adversely. In our study, patients with ER of T1/Tis stage tumors had significantly lower occurrence rates of MAA. Combining ER and surgery could improve the discovery rate of SCRC and offer significant surveillance colonoscopy benefits. The treatment strategy in patients with SCRC is suggested in Figure 4.

Several previous studies^17,21,22^ have shown that solitary CRC patients with SA or SAA (which is usually defined as adenoma with villous histology, high-grade dysplasia, or size ≥10 mm) have a significantly increased risk of metachronous neoplasms in colonoscopy, especially those with high-grade intraepithelial neoplasia. The possible reason is that the tumor susceptibility gene still promotes tumor lesions after colorectal cancer resection. The presence of SA and SAA at baseline colonoscopy was significantly associated with the development of MA in our study. The detection of SA was associated independently with an increased risk of having MA at follow-up evaluation (OR = 15.0; 95% CI: 1.55-145.22). We argued that SCRC patients in the presence of synchronous neoplasms should be given in the postoperative follow-up colonoscopy more closely for the early detection of metachronous neoplasms.

According to the National Comprehensive Cancer Network Guidelines,^23^ enhanced CT is the preferred diagnostic method for colorectal cancer evaluating distant metastasis after surgery. Survival rates are higher in CRC patients who undergo CT than in those who do not undergo these investigations.^24^ However, intensive surveillance with CT does not improve overall survival and recurrence-free survival in a retrospective observational study.^25^ Thus, doctors should seek the balance between terms of oncologic benefit and medical economics. In our study, distant metastasis was found in approximately a quarter of the patients (23.6%), but a relationship was not found between endoscopic and radiological findings. One possible explanation was that the sample size is not large enough to be significant.

There were a few limitations associated with our study. First, as a single-center, retrospective, and prospective cohort study, selection bias was inevitable. Second, we did not take into account the impact of molecular biology (microsatellite instability, p53 mutation, or K-ras mutation). Considering that the molecular biology of SCRC tumors is not completely consistent with solitary colorectal carcinoma,^2^ large-sample clinical research should be carried out to derive more precise conclusions. Despite these limitations, our study met with an important need that there have been few studies on the surveillance colonoscopy of SCRC to date.

## Conclusion

For postoperative SCRC patients, individual surveillance strategies should be designed according to sex, baseline colonoscopy, and operative (or endoscopic) approach of resection.

## Figures and Tables

**Table 1.** Anatomical (a) Distribution and Stage (b) of First, Second, and Third Tumors in Patients with SCRCs (n = 38)

Tumor Location Index, Second and Third Tumor	n (%)	Tumor Stage Index, Second and Third Tumor	n (%)
(a)		(b)	
Right sided - Right sided	2 (5.3)	III/IV-Tis	5 (13.2)
Right sided - Left sided	10 (26.3)	I/II-Tis	3 (7.9)
Left sided - Left sided	6 (15.8)	Tis-Tis	1 (2.6)
Left sided - Right sided	3 (7.9)	IV-IV	2 (5.3)
Left sided - Rectum	4 (10.5)	IV-III	4 (10.5)
Rectum - Left sided	5 (13.2)	IV-II	3 (7.9)
Rectum - Left sided	2 (5.3)	IV-I	4 (10.5)
Rectum - Rectum	1 (2.6)	III-III	5 (13.2)
Right sided - Right sided - Right sided	1 (2.6)	III-II	4 (10.5)
Right sided - Right sided - Rectum	1 (2.6)	II-II	2 (5.3)
Rectum - Cecum - Right sided	1 (2.6)	IV-IV-Tis	2 (5.3)
Left sided - Left sided - Left sided	1 (2.6)	IV-I-I	1 (2.6)
Rectum - Left sided - Left sided	1 (2.6)	III-III-II	2 (5.3)

**Table 2.** Comparison of Pathological Findings Between Index Lesions and Concurrent Lesions in SCRC

	Index Lesion (n = 38)	Concurrent Lesion (n = 43)	* P*
Differentiation			
Highly (n = 30)	5 (13.2)	25 (58.1)	<.001
Moderately (n = 36)	22 (57.9)	14 (32.6)	
Poorly (n = 7)	5 (13.2)	2 (4.7)	
Mucinous (n = 8)	6 (15.8)	2 (4.7)	
Tumor location			
Right-sided cancer (n = 24)	14 (36.8)	10 (23.3)	.064
Left-sided cancer (n = 41)	14 (36.8)	27 (62.8)	
Rectum cancer (n = 16)	10 (26.3)	6 (13.9)	
Tumor stage			
Tis (n = 12)	1 (2.6)	11 (25.6)	<.001
I (n = 8)	2 (5.3)	6 (13.9)	
II (n = 14)	3 (7.9)	11 (25.6)	
III (n = 25)	14 (36.8)	11 (25.6)	
IV (n = 22)	18 (47.4)	4 (9.3)	
Morphology			
Elevated (n = 57)	17 (44.7)	40 (93.0)	<.001
Ulcerated (n = 24)	21 (55.3)	3 (7.00	
Lymphatic invasion			
No (n = 63)	22 (57.9)	41 (95.3)	<.001
Yes (n = 18)	16 (42.1)	2 (4.7)	
Venous invasion			
No (n = 72)	30 (78.9)	42 (97.7)	.011
Yes (n = 9)	8 (21.1)	1 (2.3)	
Nerve invasion			
No (n = 66)	25 (65.8)	41 (95.3)	.001
Yes (n = 15)	13 (34.2)	2 (4.7)	

**Table 3.** Predictors of Metachronous Adenoma and Metachronous Advanced Adenoma During Follow-up

	Any MA (n, %)	* P*	Any MAA (n, %)	*P*
Sex				
Male (n = 24)	17 (70.8)	.037	6 (33.3)	.455
Female (n = 12)	4 (33.3)		2 (16.7)	
Age	69.13 (12.60)	.047	67.00 (16.41)	.746
CEA (ng/mL)	4.86 (1.47-9339.38)	.187		.302
Tumor location				
With rectum cancer (n = 13)	8 (61.5)	.526	2 (15.4)	.382
Without rectum cancer (n = 23)	13 (56.5)		6 (26.1)	
Stage of index tumor				
Tis/I/II (n = 6)	5	.185	2	.382
III/IV (n = 30)	16		6	
Endoscopy resection				
No (n = 24)	12	.289	18	.010
Yes (n = 12)	9		3	
Synchronous adenomas				
No (n = 8)	1 (12.5)	.013	1 (12.5)	.503
Yes (n = 28)	20 (71.4)		7 (25.0)	
Synchronous advanced adenomas				
No (n = 10)	3 (30.0)	.039	1 (10.0)	.269
Yes (n = 26)	18 (69.2)		7 (26.9)	

MA, metachronous adenoma; MAA, metachronous advanced adenoma.
